# Combined detection of serum EFNA1 and MMP13 as diagnostic biomarker for gastric cancer

**DOI:** 10.1038/s41598-024-65839-y

**Published:** 2024-07-10

**Authors:** Ling-Yu Chu, Fang-Cai Wu, Hai-Peng Guo, Jian-Jun Xie, Qi-Qi Qu, Xin-Hao Li, Yi-Wei Xu, Yu-Hui Peng, Bo Qiu

**Affiliations:** 1 Department of Pathology, Medical College of Jiaying University, No. 146 Huangtang Road, Meizhou, China; 2 Department of Clinical Laboratory Medicine, Esophageal Cancer Prevention and Control Research Center, the Cancer Hospital of Shantou University Medical College, No. 7 Raoping Road, Shantou, China; 3 Department of Biochemistry and Molecular Biology, Shantou University Medical College, Shantou, China; 4 Guangdong Esophageal Cancer Institute, The Cancer Hospital of Shantou University Medical College, Shantou, China; 5 Department of Head and Neck Surgery, Esophageal Cancer Prevention and Control Research Center, The Cancer Hospital of Shantou University Medical College, Shantou, China; 6 Department of Radiation Oncology, Esophageal Cancer Prevention and Control Research Center, The Cancer Hospital of Shantou University Medical College, Shantou, China

**Keywords:** Gastric cancer, EFNA1 and MMP13, Diagnosis, Serum biomarker, Early detection, Cancer, Molecular biology, Biomarkers, Gastroenterology, Oncology

## Abstract

We previously identified that serum EFNA1 and MMP13 were potential biomarker for early detection of esophageal squamous cell carcinoma. In this study, our aim is to explore the diagnostic value of serum EFNA1 and MMP13 for gastric cancer. We used enzyme-linked immunosorbent assay (ELISA) to detect the expression levels of serum EFNA1 and MMP13 in 210 GCs and 223 normal controls. The diagnostic value of EFNA1 and MMP13 was evaluated in an independent cohorts of GC patients and normal controls (n = 238 and 195, respectively). Receiver operating characteristics were used to calculate diagnostic accuracy. In training and validation cohorts, serum EFNA1 and MMP13 levels in the GC groups were significantly higher than those in the normal controls (P < 0.001). The area under the curve (AUC) of the combined detection of serum EFNA1 and MMP13 for GC was improved (0.794), compared with single biomarker used. Similar results were observed in the validation cohort. Importantly, the combined measurement of serum EFNA1 and MMP13 to detect early-stage GC also had acceptable diagnostic accuracy in training and validation cohort. Combined detection of serum EFNA1 and MMP13 could help identify early-stage GC, suggesting that it may be a promising tool for the early detection of GC.

## Introduction

Gastric cancer (GC) is one of the most common and deadly malignant tumors in the world. Although morbidity and mortality are declining worldwide, GC is still the fourth leading cause of cancer deaths^1^. In recent years, there have been for over one million new cases of GC, making it the fifth most common malignant tumor in the world^1^. Among them, cases reported in China account for a large proportion^2,3^. It is reported that the long-term survival rate of patients with advanced GC was less than 30%^4^. Studies have shown that the 5-year survival rate of patients with early stage of GC exceeds 90%, who were treated by endoscopic submucosal dissection^5^. Consequently, the identification of effective approaches to detect early stage GC is the key to improving GC patients survival.

Early detection of tumors is the most desirable approach in the management of GC. Noteworthily, the conventional serological tumor markers such as carcinoembryonic antigen (CEA), carbohydrate antigen (CA) 19-9 and CA72-4 are inadequate for detecting GC due to their poor sensitivity and specificity^6–8^. Although endoscopy combined with pathological results is the gold standard for the diagnosis of GC, its acceptability is poor and it is difficult to be used for large-scale clinical screening^9,10^. Therefore, it is necessary to explore potential biomarkers related to GC and try to bring new ideas to the research of early diagnosis and detection, targeted therapy, and long-term monitoring of GC. In this regard, cell-free proteins in the serum have the potential to be a safer diagnostic biomarker in GC.

A large number of studies have shown that EFNA1 is often overexpressed in human gastrointestinal tumors such as CRC, EC and HCC, and the degree of up-regulation of EFNA1 is closely related to tumor malignancy, metastasis potential and patient prognosis^11,12^. In addition, studies have also shown that MMP13 is overexpressed in tumors, such as nasopharyngeal carcinoma, cutaneous squamous cell carcinoma, breast cancer, and head and neck squamous cell carcinoma, making it a potential diagnostic and therapeutic target^13^. MMP13 is also a key factor in tumor tissue invasiveness, metastasis, and prognosis^14^. EFNA1 and MMP13, as secreted proteins, are overexpressed in many cancers, but their expression in normal tissues is very low or undetectable, suggesting their potential as serum diagnostic markers. Therefore, this article explored the diagnostic efficacy of a new serological marker EFNA1 combined with MMP13 for early GC.

EFNA1 is a membrane protein anchored on the cell surface through glycosylphosphatidylinositol (GPI) bonds^15^. It is a protein product secreted by human umbilical vein endothelial cells (HUVECs) stimulated by tumor necrosis factor (TNF). EFNA1 binds to many Eph A receptors (EphA1-5)^16,17^ and is a good in vitro molecular marker of endothelial cells^18^. However, the diagnostic value of serum EFNA1 for GC has not been confirmed. As a member of Matrix metalloproteinases (MMPs), MMP13 belongs to a type of collagenase. Its main physiological role is to destroy type II collagen and plays an important role in extracellular matrix circulation, cancer cell migration, cell growth, inflammation, and angiogenesis^19,20^. MMP13 was first discovered in breast cancer, and more and more researches have been conducted in the diagnosis of bladder cancer, colorectal cancer, cervical cancer, and other common tumors^21–23^. However, there are few studies on MMP13 in the diagnosis of GC, especially in serology.

We have recently reported that an integrated Five-Biomarker Panel (iFBPanel) (EFNA1, MMP13, CEA, Cyfra21-1 and squmaous cell carcinoma antigen) might be used as a blood biomarker-based tool to identify early-stage esophageal squamous cell carcinoma (ESCC)^24^. Study found that serum EFNA1 and MMP13 levels in early-stage and all-stage ESCC patients were significantly higher than those in normal controls^24^. In addition, the diagnostic performance of EFNA1 combined with MMP13 was superior to that of EFNA1 or MMP13 alone. And this is similar to the data we found in the GC, which provided evidences for serum EFNA1 combined with MMP13 may be a biomarker for diagnosis of GC.

## Results

### Screening of EFNA1 and MMP13 in GC

First, by the integrated analysis of ChIP-Seq, RNA-sequence, secretome data, GEO databases and measurement of a small size of clinical samples by ELISA, serum EFNA1 and MMP13 were identified as secreted proteins encoded by super enhancer driven genes^24^. Then, we analyzed the levels of EFNA1 and MMP13 in GC cell lines and tissues and their relationships with the prognosis of GC patients through transcriptional open data sets (CCLE, GEPIA and Kaplan–Meier Plotter). Compared with other cancers, EFNA1 and MMP13 were highly or moderately expressed in GC (Supplementary Fig. S1A). Moreover, according to TCGA data, we found that EFNA1 and MMP13 were up-regulated in GC (Supplementary Fig. S1B). After Kaplan–Meier analysis and log-rank test, EFNA1 and MMP13, were identified to show prognostic value in GC (Supplementary Fig. S1C). Based on the comprehensive analysis of the above results, EFNA1 and MMP13 were screened as potential diagnostic biomarkers for GC.

### The levels of serum EFNA1 and MMP13 in GC patients and normal controls

**Figure 1:**
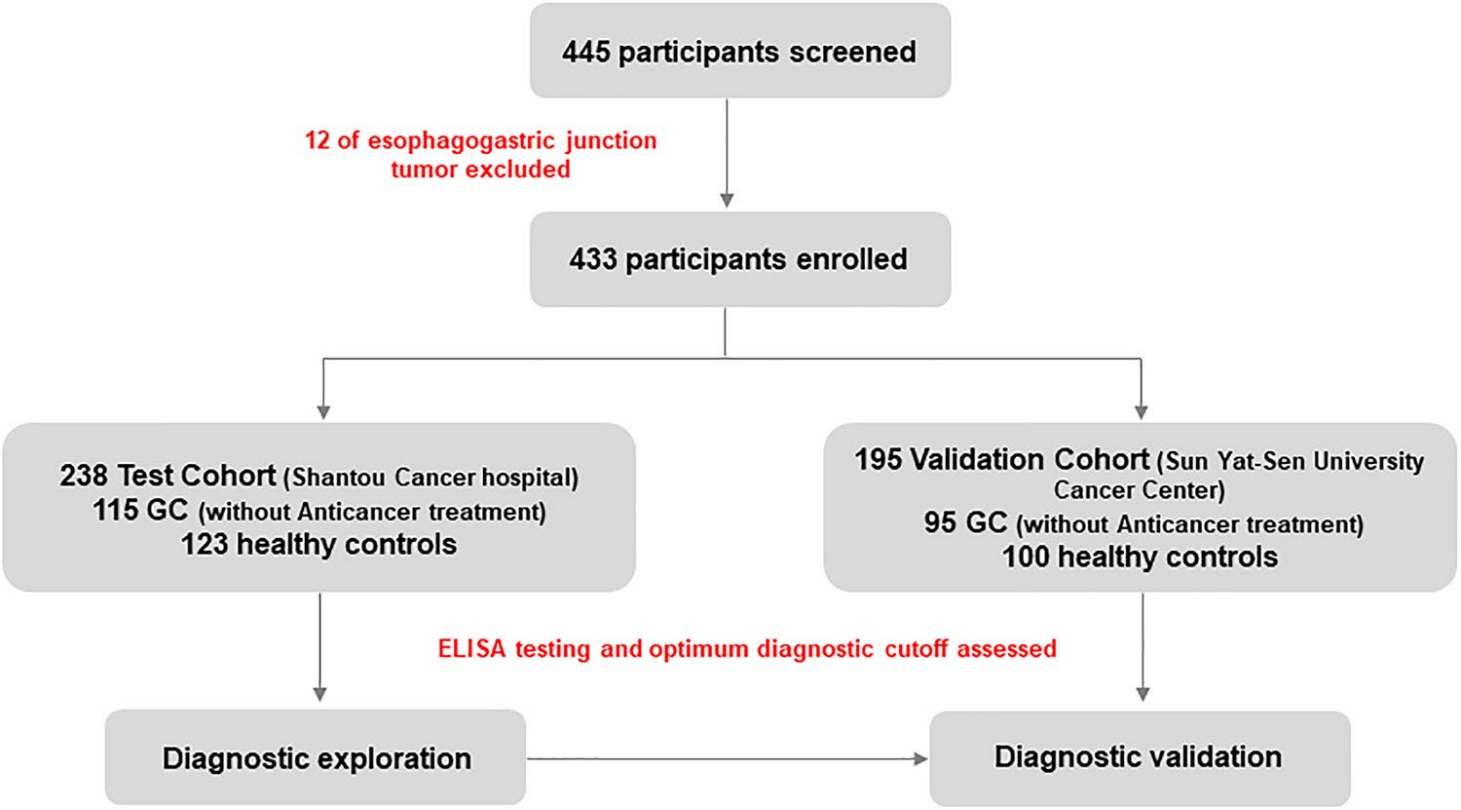
Study profile. *GC* gastric cancer, *ELISA* enzyme-linked immunosorbent assay.

**Figure 2:**
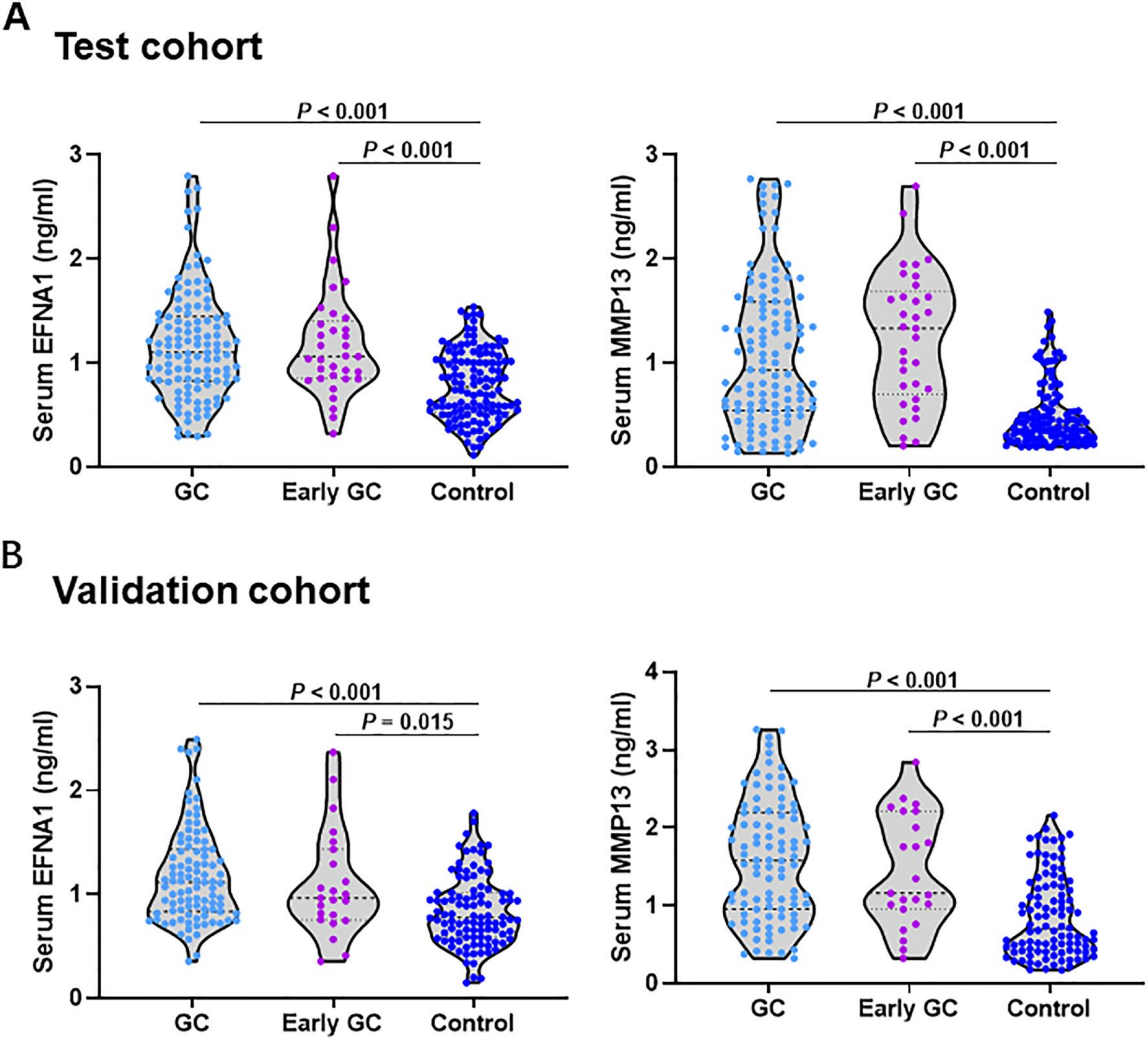
The concentration of serum EFNA1 and MMP13 shown in scatter plots and violin plots. (**A**) OD values of EFNA1 and MMP13 from GC sera (115) and normal sera (123) in the test cohort. (**B**) OD values of EFNA1 and MMP13 from GC sera (95) and normal sera (100) in the validation cohort. *GC* gastric cancer, *ELISA* enzyme-linked immunosorbent assay.

Figure 1 shows that the study recruited a total of 433 participants, of which 238 were in the training cohort and 195 were in the validation cohort. Table 1 summarizes the clinicopathological characteristics of GC patients in the two cohorts. The average concentration ± standard error (SEM) of EFNA1 in GC was 1.17 ± 0.52 ng/mL, while the normal controls and early-stage GC were 0.79 ± 0.33 ng/mL and 1.18 ± 0.51 ng/mL (Table 2). The mean serum MMP13 concentration ± SEM of the GC, normal controls and early-stage GC were 1.08 ± 0.71 ng/mL, 0.45 ± 0.30 ng/mL, and 1.25 ± 0.64 ng/mL, respectively (Table 2). As shown in Fig. 2A and Table 2, the serum EFNA1 and MMP13 levels of GC patients in the training cohort were higher than those in the normal controls, which was confirmed by statistics (*P* < 0.001). The difference between early-stage GC and normal controls was also significant (*P* < 0.001). Similar results were found in the validation cohort (Fig. 2B, Table 2). Subsequently, we analyzed the expression of EFNA1 and MMP13 in serum of GC patients in test cohort and validation cohort, and we found that EFNA1 and MMP13 levels did not differ significantly between different cohort (Supplementary Fig. S2).

**Table 1:** Clinicopathological parameters of patients in training cohort (Cohort 1) and validation cohort (Cohort 2).

Group	Cohort 1				Cohort 2			
	GC (n = 115)		Normal (n = 123)		GC (n = 95)		Normal (n = 100)	
	NO	%	NO	%	NO	%	NO	%
Age, years								
Mean	57 ± 10		56 ± 10		55 ± 10		52 ± 9	
Range	26–81		30–84		31–76		40–81	
Gender								
Male	63	67	51	41.5	59	62.1	76	76
Female	52	33	72	58.5	36	37.9	24	25
TNM stage								
I	9	7.8			7	7.4		
II	24	20.9			20	21.1		
III	55	47.8			41	43.2		
IV	24	20.9			23	24.2		
Unknown	3	2.6			4	4.2		
Depth of tumor invasion								
T1	8	7			8	8.4		
T2	4	3.5			4	4.2		
T3	23	20			36	37.9		
T4	76	66.1			43	45.3		
Unknown	4	3.5			4	4.2		
Lymph node metastasis								
Positive	77	67			69	72.6		
Negative	29	25.2			21	22.1		
Unknown	9	7.8			5	5.3		
Distant metastasis								
Yes	14	12.2			21	22.1		
No	96	83,5			70	73.7		
Unknown	5	4.3			4	4.2

**Table 2:** Comparison of serum EFNA1 and MMP13 expression levels in in GC, early-stage GC and normal controls.

	N	Serum biomarker expression	*P* value*
		Mean ± SD	
Test cohort			
EFNA1			
GC	115	1.17 ± 0.52	< 0.001
Early-stage GC	33	1.18 ± 0.51	< 0.001
Normal controls	123	0.79 ± 0.33	
MMP13			
GC	115	1.08 ± 0.71	< 0.001
Early-stage GC	33	1.25 ± 0.64	< 0.001
Normal controls	123	0.45 ± 0.3	
Validation cohort			
EFNA1			
GC	95	1.18 ± 0.45	< 0.001
Early-stage GC	23	1.10 ± 0.51	0.015
Normal controls	100	0.84 ± 0.34	
MMP13			
GC	95	1.19 ± 0.60	< 0.001
Early-stage GC	23	1.07 ± 0.53	< 0.001
Normal controls	100	0.64 ± 0.39

### Serum EFNA1 and MMP13 can be used as an early-diagnosis biomarker for patients with GC

**Figure 3:**
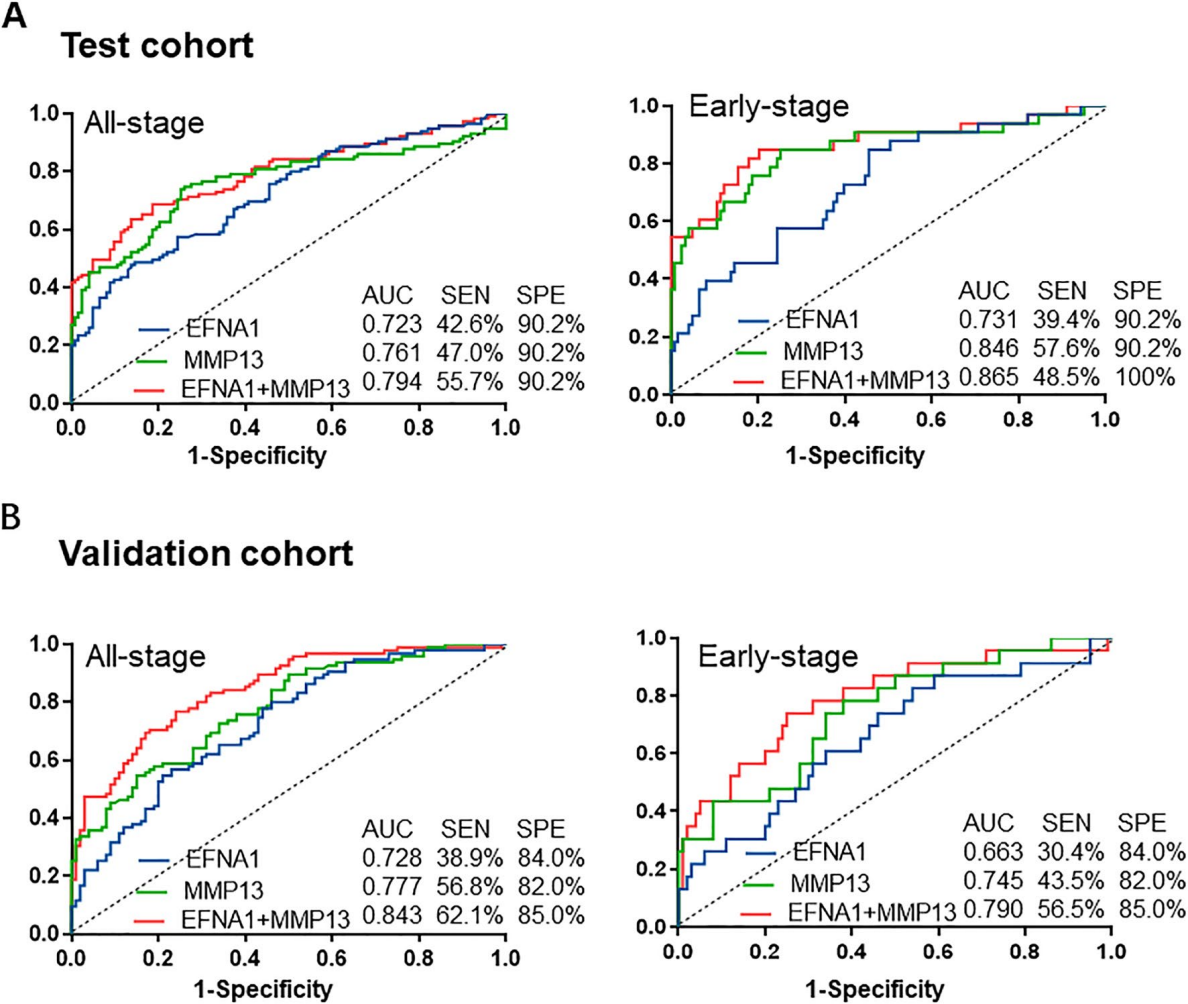
ROC curve analysis in the diagnosis of all stages and early-stage GC. (**A**) The performance of the EFNA1, MMP13 and combination of EFNA1 and MMP13 in distinguishing all stages and early-stage ESCC from normal controls are shown for the test cohort. (**B**) The performance of the EFNA1, MMP13 and combination of EFNA1 and MMP13 in distinguishing all stages and early-stage GC from normal controls are shown for the validation cohort. The area under the black line is 0.5, for reference. *ROC* Receiver operating characteristic, *GC* gastric cancer.

The serum EFNA1 and MMP13 concentrations in patients with early-stage GC (TNM stage 0 + I + IIA) are similar to those in patients with all-stage GC, and both are significantly higher than those of normal controls (Table 2). Therefore, we tried to evaluate the diagnostic value of serum EFNA1 and MMP13 in patients with early-stage GC. ROC curve analysis indicated that the optimized cutoff values for EFNA1 and MMP13 to distinguish between GC and normal controls were 1.21 ng/ml and 1.03 ng/ml, respectively. In addition, the diagnostic efficiency of serum EFNA1 combined with MMP13 (AUC 0.794, sensitivity 55.7%, specificity 90.2%) in GC patients was significantly higher than the two single tests (EFNA1 (AUC 0.723, sensitivity 42.6%, specificity 90.2%), MMP13 (AUC 0.761, sensitivity 47.0%, specificity 90.2%)) (Fig. 3A and Table 3). In patients with early-stage GC, we also observed the diagnostic ability of serum EFNA1 combined with MMP13 to distinguish cancer patients from normal controls (AUC 0.865, sensitivity 48.5%, specificity 90.2%) (Fig. 3A and Table 3, Supplementary excel). Similar results were obtained in the comparison between GC patients, early-stage GC patients, and normal controls in the validation cohort (Fig. 3B and Table 3, Supplementary excel). These results together prove that serum EFNA1 combined with MMP13 can be used as a good biomarker for detecting early-stage GC.

**Table 3:** Results for measurement of EFNA1, MMP13 and EFNA1 + MMP13 in the diagnosis of GC and early-stage GC.

Group	AUC (95%CI)	SEN	SPE	FPR	FNR	PPV	NPV	PLR	NLR
Test cohort									
All-stage									
EFNA1 vs. NC	0.723 (0.659–0.787)	42.6%	90.2%	9.8%	57.4%	80.3%	62.7%	4.37	0.64
MMP13 vs. NC	0.761 (0.697–0.825)	47.0%	90.2%	9.8%	53.0%	81.8%	64.5%	4.81	0.59
EFNA1 + MMP13 vs. NC	0.794 (0.736–0.852)	55.7%	90.2%	9.8%	44.3%	84.2%	68.5%	5.70	0.49
Early-stage									
EFNA1 vs. NC	0.731 (0.635–0.827)	39.4%	90.2%	9.8%	60.6%	52.0%	84.7%	4.04	0.67
MMP13 vs. NC	0.846 (0.757–0.934)	57.6%	90.2%	9.8%	42.4%	61.3%	88.8%	5.90	0.47
EFNA1 + MMP13 vs. NC	0.865 (0.781–0.949)	48.5%	90.2%	9.8%	51.5%	57.1%	86.7%	4.97	0.57
Validation cohort									
All-stage									
EFNA1 vs. NC	0.728 (0.659–0.798)	38.9%	84.0%	16.0%	61.1%	68.5%	58.9%	2.29	0.74
MMP13 vs. NC	0.777 (0.714–0.841)	56.8%	82.0%	18.0%	43.2%	74.0%	66.4%	2.99	0.53
EFNA1 + MMP13 vs. NC	0.843 (0.789–0.897)	62.1%	85.0%	15.0%	37.9%	79.7%	70.2%	4.14	0.45
Early-stage									
EFNA1 vs. NC	0.663 (0.537–0.790)	30.4%	84.0%	16.0%	69.6%	29.2%	83.8%	1.79	0.84
MMP13 vs. NC	0.777 (0.633–0.856)	43.5%	82.0%	18.0%	56.5%	34.5%	86.2%	2.29	0.70
EFNA1 + MMP13 vs. NC	0.843 (0.680–0.901)	56.5%	85.0%	15.0%	43.5%	46.4%	89.5%	3.77	0.51

*AUC* area under the curve, *95% CI* 95% confidence interval, *GC* gastric cancer, *NC* normal controls, *SEN* Sensitivity, *SPE* Specificity, *FNR* false negative rate, *FPR* false positive rate, *PPV* positive predictive value, *NPV* negative predictive value, *PLR* positive likelihood ratio, *NLR* negative likelihood ratio.

### Correlation between serum concentration of EFNA1/MMP13 and clinicopathological features

The relationship between serum EFNA1, MMP13 levels, and clinicopathological characteristics is shown in Table 4. The positive rate of serum EFNA1 + MMP13 was not significantly correlated with clinical data such as age, gender, depth of tumor invasion, lymph node status, TNM stage, early-stage or late-stage GC (all *P* > 0.05). The validation cohort also got similar results.

**Table 4:** Correlation between EFNA1 + MMP13 and clinicopathologic characteristics of GC patients in both test and validation groups.

Group	Test cohort			Validation cohort		
	n	Positive (%, 95% CI)	*P*	n	Positive (%, 95% CI)	*P*
Age, years						
≤ 55	37	18 (48.6, 32.2–65.3)	0.321	43	26 (60.5, 44.5–74.6)	0.833
> 55	78	46 (58.9, 47.3–69.8)		52	33 (63.5, 48.9–76.0)	
Gender						
Male	63	39 (61.9, 48.8–73.6)	0.187	59	37 (62.7, 49.1–74.7)	0.876
Female	52	25 (48.1, 34.2–62.2)		36	22 (61.1, 43.5–76.4)	
TNM stage						
I	9	6 (66.7, 30.9–91.0)	0.722	7	4 (57.1, 20.2–88.2)	0.375
II	24	15 (62.5, 40.8–80.4)		20	9 (45.0, 23.8–68.0)	
III	55	27 (49.1, 35.5–62.8)		41	26 (63.4, 46.9–77.4)	
IV	24	14 (58.3, 36.9–77.2)		23	17 (73.9, 51.3–88.9)	
Unknown	3	2 (66.7, 12.5–98.2)		4	3 (75.0, 21.9–98.7)	
Depth of tumor invasion						
T1	8	7 (87.5, 46.7–99.3)	0.292	8	5 (62.5, 25.9–89.8)	0.331
T2	4	1 (25.0, 1.30–78.1)		4	2 (50.0, 9.20–90.8)	
T3	23	13 (56.5, 34.9–76.1)		36	18 (50.0, 33.2–66.8)	
T4	76	41 (53.9, 42.2–65.3)		43	31 (72.1, 56.1–84.2)	
Unknown	4	2 (50.0, 9.20–90.8)		4	3 (75.0, 21.9–98.7)	
Lymph node metastasis						
Positive	77	40 (51.9, 40.3–63.4)	0.508	69	45 (65.2, 52.7–76.0)	0.242
Negative	29	18 (62.1, 42.4–78.7)		21	10 (47.6, 26.4–69.7)	
Unknown	9	6 (66.7, 30.9–91.0)		5	4 (80.0, 29.9–98.9)	
Early-stage vs. Late-stage						
Early (0 + I + IIA)	33	21 (63.6, 45.1–79.0)	0.484	23	12 (55.2, 31.1–72.6)	0.343
Late (IIB + III + IV)	79	41 (51.9, 40.4–63.2)		68	44 (64.7, 52.1–75.6)	
Unknown	3	2 (66.7, 12.5–98.2)		4	3 (75.0, 21.9–98.7)	

*GC* gastric cancer. *95% CI* 95% exact confidence interval. Statistical significance was determined by means of Chi-squared test.

## Discussion

There have been efforts to develop non-invasive biomarkers for cancer diagnosis and treatment. Cancer cells are characterized by rapid growth, invasion, and metastasis with an abundant blood supply, which results in the constant release of tumor cells into the bloodstream. In this regard, blood-based biomarkers can reflect the real-time biological characteristics of tumors and have been recognized as emerging indicators for diagnosing cancer, detecting recurrence^25–27^, or monitoring treatment response of several malignant tumors^28,29^. Overall, this highlights the importance of developing a blood-based biomarker for the diagnosis of patients with GC.

In this study, we examined the levels of EFNA1 and MMP13 in sera from GC patients and normal controls, and the analysis revealed that EFNA1 and MMP13 were potential diagnostic biomarkers for GC. The EFNA1 combined with MMP13 demonstrated acceptable accuracy in the diagnosis of GC, especially for early-stage patients. The diagnostic values of EFNA1 combined with MMP13 were verified in the training cohort of 115 patients and 123 controls and in the independent validation cohort of 95 patients and 100 controls.

High sensitivity is an important indicator to avoid false-negative diagnoses. Herein, the optimized cut-off values ​​we determined for EFNA1 and MMP13 are 1.21 ng/ml and 1.03 ng/ml, respectively. The sensitivities of EFNA1 and MMP13 at the optimal critical level were 42.6% and 47.0%, respectively, while the sensitivity of the combined application of the two markers increased to 55.7%. And the data were further verified in the validation cohort. Moreover, EFNA1 combined with MMP13 potentially demonstrated a better diagnostic sensitivity for early-stage GC patients than markers CEA and CA19-9, which are major serum tumor markers in gastrointestinal cancers currently used in clinical practice. The positive rates of CEA and CA19-9 in GC patients were reported to be only 25.5% and 38.7%, respectively, and these markers are elevated most commonly in advanced-stage patients^30,31^. In addition, the AUC of EFNA1 and MMP13 for diagnosing GC were 0.723 and 0.761, respectively, while the AUC of GC using both two tumor markers was 0.794. The robustness of the diagnostic features was confirmed in two independent cohorts. The diagnostic efficiency for early-stage GC was in accordance with our previous studies on assessing EFNA1 combined with MMP13 for early-stage ESCC^24^. This result suggests that if asymptomatic population are detected with positive result of EFNA1 combined with MMP13, they should be considered at higher risk for suffering from GC or ESCC.

As a member of the EFN family, EFNA1 widely participates in tumorigenesis by influencing tumor angiogenesis and malignant cell phenotypes. In GC, a higher expression of EFNA1 was found in most samples, and its expression was related to tumor stage, depth of invasion, lymph node metastasis, and recurrence^32^. In addition, EFNA1 was detected in the supernatant of most of the authentic Hepatocellular carcinoma (HCC) cell lines, and elevated serum EFNA1 levels were noted for HCC patients by comparing to the patients with liver cirrhosis^33^. Moreover, our previous studies also showed that the expression of serum EFNA1 in ESCC was significantly higher than that of normal controls indicating EFNA1 as a novel serum marker for the detection of cancers. Here, we showed the potential utility of EFNA1 in the early diagnosis of GC which furtherly confirms the crucial role of soluble EFNA1 in the progression of tumors. MMPs are matrix enzymes belonging to the zinc-calcium-dependent family of endopeptidases. It can cleave ephrinA1, suggesting a biological relevance of EFNA1 and MMP13^34^. MMP13 plays a role in the degradation of basement membrane and extracellular components, destroys the histological barrier of tumor invasion, and promotes tumor invasion and migration^20^. In GC, MMP13 was reported to be up-regulated in tumor tissues and its positive expression was related to poorer survival^35^. In this study, we further revealed the higher level of MMP13 in the serum of GC patients by comparing it to the normal control and MMP13 might serve as a biomarker for early-stage GC.

Although our study shows for the first time that the detection of serum EFNA1 combined with MMP13 can play an auxiliary role in the diagnosis of GC, there are still some limitations in this study. Firstly, this prospective study selected relatively simple tissue types (only gastric adenocarcinoma). Therefore, to further verify the role of EFNA1 combined with MMP13 in the diagnosis of GC, it is necessary to conduct retrospective studies on multi-centers, large samples, and multiple tissue types [such as gastric malt and Gastrointestinal stromal tumors (GIST)]. Secondly, there are no strict restrictions on the types of gastritis control cases, and some diseases that may affect serum EFNA1 and MMP13 levels, such as infection, ischemia, and diabetes, are also not considered. In addition, changes in serum EFNA1 and MMP13 concentrations may help to dynamically monitor the prognosis of GC patients undergoing surgical treatment. However, due to the small number of cases, we did not evaluate the changes in the expression levels of serum EFNA1 and MMP13 before and after surgery in GC patients. This study did not further detect the correlation between EFNA1 and MMP13 expression levels in GC tissues and serum expression levels. This is indeed a point where we have not considered morality, and it is the direction we need to work on in the future. Finally, we only tested the diagnostic efficacy of EFNA1 combined with MMP13 for ESCC and GC. Since most of the tumor markers identified so far are not specific to a particular tumor type, we can speculate that EFNA1 combined with MMP13 may also have potential diagnostic value for other tumor types, which needs future evaluation.

In conclusion, our study shows that the levels of serum EFNA1 and MMP13 in GC patients are significantly increased. In addition, the combined detection of EFNA1 and MMP13 significantly improves the diagnostic efficiency of GC and early-stage GC, indicating that EFNA1 combined with MMP13 may be an independent tumor marker for GC patients, and the combined detection of the two tumor markers is helpful for the diagnosis of GC.

## Materials and methods

### Study samples

In this study, we selected the examination information of patients who were examined in the Cancer Hospital of Shantou University Medical College from June 2013 to May 2019 and included 115 GC patients and 123 healthy controls as the training cohort for research. In addition, 95 GC patients and 100 healthy controls collected from March 2018 to September 2019 in the Sun Yat-Sen University Cancer Center were selected as the validation cohort. The patients included in the analysis met the following criteria: (1) All patients meet the diagnostic criteria for primary GC and were diagnosed as GC by histopathological examination; (2) GC patients did not have any cancer or received any anti-cancer treatment before diagnosis; (3) Their clinical data is complete. All healthy controls were qualified blood donors without previous malignant diseases. In this study, all serum results were obtained before the start of treatment. The collected serum samples were coagulated at room temperature for 30 min, and then centrifuged at 1250*g* for 5 min. Finally, store them in a refrigerator at − 80 °C until the start of the experiment.

According to the eighth edition of the American Joint Committee on Cancer (AJCC) Cancer Staging Manual^36^, we have staged GC, in which AJCC stage 0 + I + IIA tumors are defined as early-stage GC. This study was approved by the Ethics Committee of Cancer Hospital of Shantou University Medical College and Sun Yat-Sen University Cancer Center, and the informed consent of all participants was obtained. All work is in line with the principles of the Declaration of Helsinki.

### Analysis of expression in cancer cell line encyclopedia (CCLE)

We downloaded the expression data of these proteins in various cell lines from CCLE and analyzed their expression differences in GC and other tumor cell lines.

### Analysis of expression and survival in public data set

We used GEPIA (http://gepia.cancer-pku.cn/) to analysis EFNA1 and MMP13 expression in GC tissues. Then, we are with Kaplan–Meier Plotter online website (http://kmplot.com/analysis/) analysis of the two kinds of protein's relationship with the prognosis of patients with GC.

### Enzyme-linked immunosorbent assay (ELISA) for EFNA1 and MMP13

ELISA kit was used to detect the expression levels of serum EFNA1(Cusabio, CSB-EL007460HU) and MMP13 (Cusabio, CSB-E04674h). We prepared reagents, samples, and standards following the manufacturer's instructions. Through preliminary experiments, we determined that the best dilution ratio of serum EFNA1 and MMP13 is 1:2 and 1:6. Then, add 100 μl of standards and samples to each well of a 96-well plate and incubate at 37 °C for 2 h. Discard the liquid in the well, add 100 μl of biotin antibody (1×) to the well plate, and incubate at 37 °C for 1 h. Wash the plate 3 times with a microplate washer (Thermo Fisher Scientific), then add 100 μl HRP avidin (1×) to each well and incubate at 37 °C for 1 h. Next, after washing 5 times, 90 μl of TMB substrate was added to each well and then incubated at 37 °C for 20 min. Add 50 μl stop solution to stop the color reaction, and read the optical density (OD) values at 450 nm and 590 nm wavelengths on the plate microplate reader (Thermo Fisher Scientific). Use the standard curve method to convert the measured OD value into a concentration, and then multiply it by the sample dilution factor to obtain the final concentration of serum EFNA1 and MMP13. Each serum sample is in duplicate, and the average is taken for analysis.

### Statistical analysis

We used the non-parametric Mann–Whitney U test to compare the expression levels of EFNA1 and MMP13 in serum of GC patients and normal controls. The results are expressed as mean ± standard deviation. The receiver operating characteristic (ROC) curve was used to calculate the area under the ROC curve (AUC) and 95% confidence interval (CI) of each group. When the specificity is > 90%, we select the optimum cut-off values by maximizing the sensitivity of the curve coordinates and minimizing the distance from the corresponding point in the ROC curve to the upper left corner. The specificity of > 90% was chosen to produce a detection method that is economical, feasible, and suitable for early detection^37^. We calculated the sensitivity, specificity, positive predictive value (PPV), and negative predictive value (NPV) (95% confidence interval [CI]) of EFNA1, MMP13, and EFNA1 + MMP13 in diagnosing GC to evaluate the diagnostic efficacy. We used the chi-square test to compare the relationship between the expression level of EFNA1 + MMP13 and clinical characteristics in the training cohort and the validation cohort. The significance of all analyses is *P* < 0.05.

We used logistics regression analysis to fit EFNA1 and MMP13 into one variable. In the test cohort, we calculate it according to the following formula: ln ($\frac{p}{1-p}$) = − 2.95 + 1.552X_1_ + 1.967X_2_; In the validation cohort, we calculate it according to the following formula: ln ($\frac{p}{1-p}$) =  − 4.36 + 2.284X_1_ + 2.317X_2_ (X_1_ is the serum expression level of EFNA1 in GC, X_2_ is the serum expression level of MMP13 in GC).

### Ethics approval

Approval of the research protocol by an institutional review board: Research involving human subjects complied with all relevant national regulations, institutional policies and is in accordance with the tenets of the Helsinki Declaration (as revised in 2013), and has been approved by the Ethics Review Committee at the Cancer Hospital of Shantou University Medical College (2015042419) and the School of Medicine and Clinical Research Ethics Committee of Sun Yat-sen University Cancer Centre (GZR2015-015).

### Consent to participate

Informed consents were obtained from all individuals included in this study.

### Supplementary Information

Supplementary Information.

Supplementary Figures.

## Data Availability

The datasets used and/or analysed during the current study are available from the corresponding author on reasonable request.
