# Supplementary material for: Combined detection of serum EFNA1 and MMP13 as diagnostic biomarker for gastric cancer
Source: Sci Rep. 2024 Jul 10;14:15957. doi: 10.1038/s41598-024-65839-y (PMC11237037; doi:10.1038/s41598-024-65839-y)
Supplement: Supplementary file 2 — Supplementary Figures. [file 41598_2024_65839_MOESM2_ESM.docx]

**SUPPLEMENTARY MATERIALS**

Ling-Yu Chu et al. “Combined detection of Serum EFNA1 and MMP13 as diagnostic biomarker for gastric cancer”

**Content
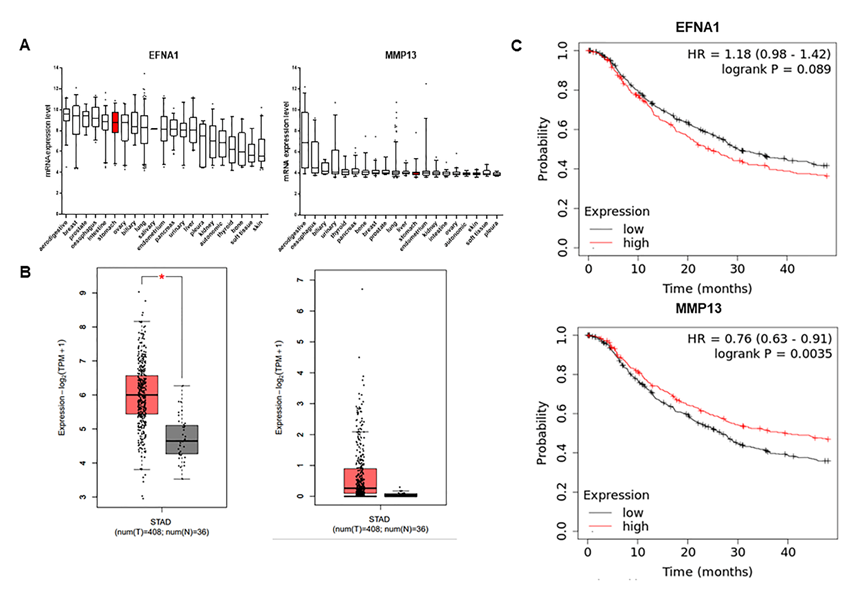
**

**Fig S1.** Screening of EFNA1 and MMP13 in GC. A. mRNA Expression levels of EFNA1and MMP13 in various tumor cell lines (Red is the expression in gastric cancer). B. Scatter plot of mRNA Expression levels of EFNA1and MMP13 in GC tissues. The black horizontal line in the box plot is the mean and the error line is SE. C. Survival curve analysis of mRNA expression levels of EFNA1and MMP13 in GC tissues.

**
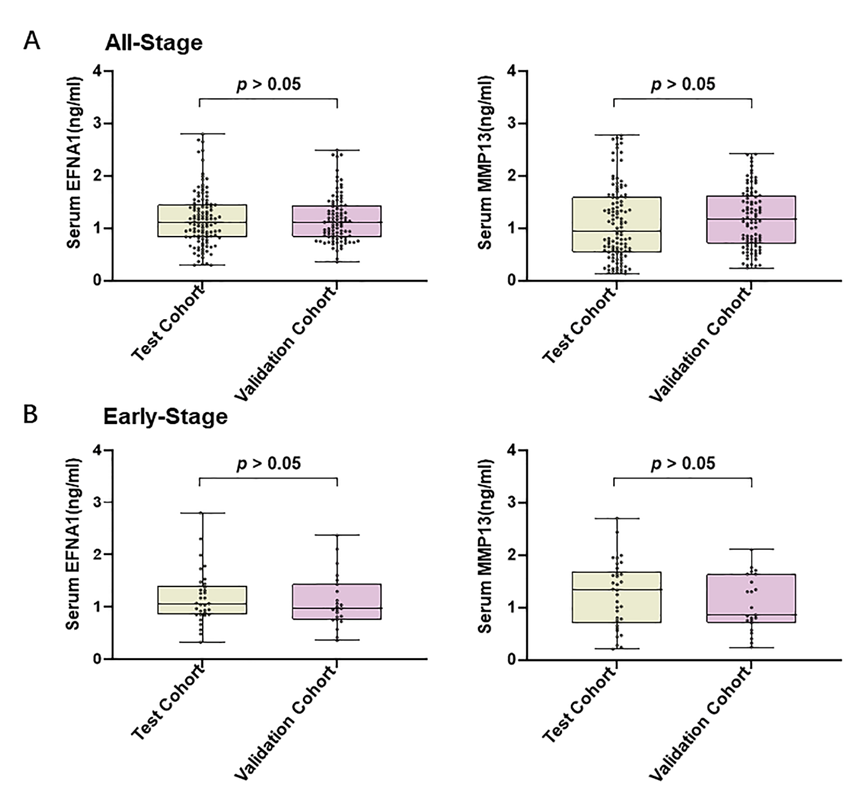
**

**Fig S2.** A. The expression levels of EFNA1 and MMP13 in serum of all-stage GC patients. B. The expression levels of EFNA1 and MMP13 in serum of early-stage GC patients. The lines in the box are means.
